# Illness recognition and appropriate care seeking for newborn complications in rural Oromia and Amhara regional states of Ethiopia

**DOI:** 10.1186/s12887-018-1196-6

**Published:** 2018-08-06

**Authors:** Y. Amare, S. Paul, L. M. Sibley

**Affiliations:** 1 Consultancy for Social Development, P.O. Box – 70196, Addis Ababa, Ethiopia; 2 Nell Hodgson Woodruff School of Nursing, Emory University, 1520 Clifton Road NE, 30322 Atlanta, Georgia; 3 Nell Hodgson Woodruff School of Nursing and Rollins School of Public Health, Emory University, 1520 Clifton Road NE, 30322 Atlanta, Georgia

**Keywords:** Newborn complications, Symptom recognition, Care seeking, Illness narratives

## Abstract

**Background:**

Ethiopia has made significant progress in reducing child mortality but newborn mortality has stagnated at around 29 deaths per 1000 births. The Maternal Health in Ethiopia Partnership (MaNHEP) was a 3.5-year implementation project aimed at developing a community-oriented model of maternal and newborn health in rural Ethiopia and to position it for scale up. In 2014, we conducted a case study of the project focusing on recognition of and timely biomedical care seeking for maternal and newborn complications. In this paper, we detail the main findings from one component of the case study – the narrative interviews on newborn complications.

**Methods:**

The study area, comprised of six districts in which MaNHEP had been implemented, was located in the two most populous federal regions of Ethiopia, Oromia and Amhara. The final purposive sample consisted of 16 cases in which the newborn survived to 28 days of life, and 13 cases in which the newborn died within 28 days of life, for a total sample size of 29 cases. Narrative interview were conducted with the main caregiver and several witnesses to the event. Analysis of the data included thematic content analysis and the determination of care seeking pathways and levels and timeliness of biomedical care seeking.

**Results:**

Mothers and other witnesses do recognize certain symptoms of newborn illness which they often mentioned in clusters. The majority considered the symptoms to be serious and in some case hopeless. Perceived causes were mostly natural. Forty-one percent of care seekers sought timely biomedical care in the neonatal period. Surprisingly, perceived severity did not necessarily trigger care seeking. Facilitators of biomedical care seeking included accessibility of health facilities and counseling by health workers, whereas barriers included perceived vulnerability of newborns, post-partum restrictions on movements, hopelessness, wait-and-see atttitudes, poor communication and physical inaccessibility of health facilities.

**Conclusions:**

Symptom recognition and care seeking patterns indicate the need to strengthen focused locally relevant health messages which target mothers, fathers and other community members, to further enhance access to health care and to improve referral and quality of care.

## Background

Globally, 2.6 million newborns died in 2016. In Ethiopia, as in much of the developing world, the death rate among children under 5 years of age has declined at a higher rate than among newborns which has remained around 29 deaths per 1000 live births in 2016. [1] Consequently, 48% of deaths in children under 5 in Ethiopia occur in the neonatal period. [2] The most important causes of newborn death globally are pre-term birth complications (36%), intra-partum related events (24%), sepsis or meningitis (16%) and congenital abnormalities (11%). [2] Low levels of facility delivery, poor newborn care practices and limited care seeking for complications are underlying factors behind high rates of newborn morbidity and care seeking.

The Maternal Health in Ethiopia Partnership (MaNHEP) was a 3.5-year implementation project funded by the Bill & Melinda Gates Foundation to develop a community-oriented model of maternal and newborn health in rural Ethiopia and to position it for scale up. [3] Emory University implemented MaNHEP, in collaboration with John Snow Research and Training Inc., University Research Co. LLC, and Addis Ababa University. In 2014, Emory conducted a case study of the project focusing on recognition of and biomedical care seeking for maternal and newborn complications. The Ethiopia case study, was one of six country studies that included India, Indonesia, Nigeria, Tanzania and Uganda. All case studies were framed by the Delay Model. [4] In an earlier 2016 publication, we focused on findings pertaining to illness recognition and care seeking for maternal complications. [5] In this paper, we detail the main findings on illness recognition and care seeking for newborn complications and their implications for policy, programming and research.

## Methods

### Study site

**Fig. 1:**
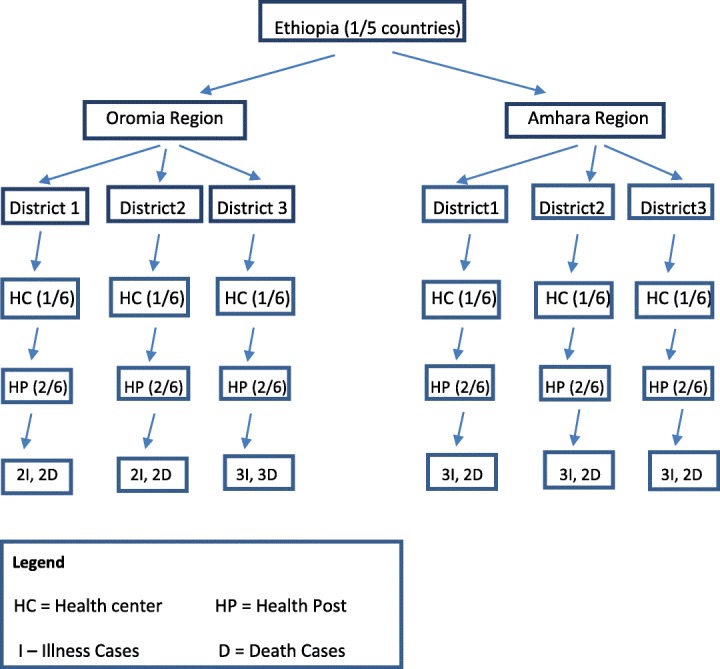
Sample design

As described in our recent publication focusing on illness recognition and care seeking for maternal complications [5], the study was conducted in the two most populous federal regions of Ethiopia, Oromia and Amhara (Fig. 1). [1] The districts were largely rural and included Degem, Kuyu and Warra Jarso in Oromia Region and North Achefer, South Achefer and Mecha in Amhara Region (estimated population 350,000). Each district has an urban center and around six health centers each of which oversee five or six health posts. From each district, one health center and two health posts were randomly selected. Cases of newborn complications occurring within the previous 6 months were identified and sampled from the catchment areas of these facilities, as described below. A case was defined as a mother, her newborn and the witnesses to the newborn’s illness event.

### Sampling and data collection

This section presents a summary of sampling and data collection procedures. For further details on sampling, the interview guide, reporting and maintainance of data quality, see our previous publication on recognition and care seeking for maternal complications [5].

In the six districts, the study aimed to involve 30 cases: for each of the six districts, 3 mothers who perceived that their newborn became ill during the first month of life and was alive at 28 days of life, 2 mothers whose newborn became ill and died within 28 days of life and several witnesses to each event . Representation of diverse views and availability of cases were considerations in sampling. The inclusion criteria for these mothers were: female, age 18–49 years, gave birth in the previous 6 months, residence in the MaNHEP project area, perceived her newborn became ill within the first month of life and willing and able to participate. The final sample consisted of 16 cases in which the newborn survived to 28 days of life, and 13 cases in which the newborn died within 28 days of life, for a total sample size of 29 cases (Fig. 1).

After obtaining verbal informed consent using standard disclosure procedures, the study team used illness narrative interviews to collect data. The illness narrative is a qualitative rendering of an illness event by those who experienced the illness, along with those who were witnesses to the event. [6]

The narrative interviews were conducted with a primary caregiver, usually the mother of the newborn, and several witnesses to the illness event, who varied in number from one-to-three additional persons including her husband, mother-in-law, mother, sibling or neighbor. Although the interviews prioritized the primary caregiver who was usually the mother, other witnesses participated to a greater or lesser extent depending on personality and their role in the management of the illness episode. Thus, it turned out that the main or only respondent(s) in 13 of the 29 interviews was the mother; the mother and her husband in seven interviews; the mother and another person such as her mother-in-law or mother in five interviews; and persons other than the mother in four interviews.

Shortly after the interviews were conducted, “expanded field notes” on them were developed from memory, field notes, and audiotape recordings.

### Analysis

. Coding procedures are detailed in our previous publication on illness recognition and care seeking for maternal complication. [5] A codebook, based on the illness narrative guide content and containing code definitions and inclusion and The analysis involved thematic content analysis using NVivo 10 based on the Delay Model [4]; re-coding of care-seeking pathways into: biomedical and non-biomedical or late biomedical categories; and univariate analysis to identify respondent characteristics and thematic code frequencies. Further details on these analyses are available in an earlier publication on care seeking for compications of pregnancy and child birth. [7] We also conducted a multiple correspondence analysis (MCA) to detect underlying structures in the illness recognition data. MCA is an exploratory qualitative data analysis technique. Perceived symptoms and causes (please refer below to the list of symptoms and causes) were treated as nominal variables with multiple levels, and the correlations among them were projected in a 2-dimensional visual “map.” Proximity between different levels of these variables and between groups of individuals associated with the levels in the map were examined for clusters or patterns of symptoms and causes in relation to outcomes. A clustering of symptoms and causes suggests illness recognition on the part of respondents. Grouping individuals by an external outcome variable allow one to examine whether clusters of symptoms and causes are associated with differential outcomes-e.g. babies survived or did not survive the first 28 days of life. MCA was performed using the statistical software R [8].

### Ethical approval

Before initiating the study, ethical review of and approval for the study was obtained from Emory University Institutional Review board and the Oromia and Amhara Regional State Health Bureaus.

## Results

### Sample characteristics

Of the 29 cases, a majority of mothers were between 19 and 29 years of age (55%) and had never attended school (62%). A majority of mothers (62%) also had given birth in a health facility. Mothers from Oromia attended more years of school than their Amhara counterparts (80% versus 43%). Of the cases, 13 cases involved newborns that had died. Of the newborns that had died, nearly all died within the first week of life (11 died day 1–3, 1 died between 4 and 7 days, and 1 died between 7 and 28 days). There were no notable differences in maternal age or education between the the group of newborns who died and those who survived. On the other hand, more babies born at home died than babies born in a health facility (eight out of the nine babies versus five of eighteen babies, respectively). The two babies who were born on the way to a health facility both survived.

### Delay 1

#### Perceived symptoms and their severity

Mothers and witnesses to the illness event mentioned a number of symptoms in their newborns. In order of frequency, many mothers mentioned inability to breastfeed (72%), followed by vomiting (41%), fever (38%), coughing, sneezing and/or stuffy nose (38%), continuous crying and weak or difficult breathing (31% each) and cold body (24%). Symptoms mentioned by between 10 and 20% of mothers included swollen uvula, weak or no crying, weakness and diarrhea. Lastly, symptoms mentioned by less than 10% of mothers included inability to pass urine or stool, change in stool color and weight loss, as well as hiccups, frothing from the mouth, bleeding from the nose and mouth, moaning, rash, swollen umbilical cord, and swelling on the back of the head and neck.

Symptoms were often mentioned in clusters. For example, eight Amhara mothers and witnesses among the total of 29 mothers noticed that the baby was unable to breastfeed, vomited and/or had a fever, high temperature on the back of the neck, in addition to a red swollen uvula. Three mothers and witnesses observed that their baby had a cough, congested nose or difficult breathing in conjunction with inability to breastfeed, fever or vomiting. Others mentioned continuous crying, inability to breastfeed, vomiting and diarrhea, along with fever and increasing weakness.

“He refused to suck my breast and when he did suck on it, he vomited soon afterwards. He cried a lot and he was sweating and had high fever. There was a sound inside his stomach when he was crying.”(Mother, Oromia).

Of the 13 babies who died, several mothers and witnesses reported that their baby cried continuously after birth, was unable to breastfeed, lost weight and had a fever. Another mother noticed that her baby felt very cold and was silent until she died, whereas a father and his mother observed that their twin babies were coughing and had a congestion, difficulty breathing and were cold. Finally, one mother and the two grandmothers realized that their twin babies were born too soon, observing that they were very small and thin, weak and/or making moaning sounds.

Seventeen of the twenty-nine mothers and witnesses believed that their newborn’s illness symptoms were serious; whereas in four cases, they thought the symptoms indicated the babies’ condition was hopeless. One woman, whose newborn experienced two separate illness episodes, perceived the initial illness to be serious, but the second illness episode as not serious.

“Yes, I was worried that the baby had fever and spent the whole night crying. I was worried that he may die. A baby cries and stops but my baby cried continuously. He also did not breastfeed and had vomiting and diarrhea.” (Mother, Amhara).

“When they [twins] came out from the womb, they were born with many problems. Even if the elder one was crying, they were coughing since birth and their body was as cold as iron. So I did not think they would start breastfeeding.” (Grandmother, Oromia).

Some mothers and witnesses also reported changing perceptions of severity as symptoms presented. In three out of the 29 cases, they thought that the symptoms were not serious or that the baby would get better. Four families believed that the symptoms were not serious, but then serious when these symptoms persisted or other symptoms appeared.

As one mother poignantly described, “I did not think that the baby was going to die. It was in the evening around nine that she was born and started to have difficulty breathing and sucking the breast. I was thinking that she may start sucking the breast next morning but she did not and her breathing problem persisted. Then she became weaker and weaker the following evening...” (Mother, Oromia).

Comparison of assessments of illness severity within cases of newborns who died versus cases of newborns who survived showed that, among the former,cases which were deemed to be hopeless were more frequent (4 of 13 cases versus 0 of 16 cases, respectively), cases which were deemed to be initially or ultimately not serious were more frequent (5 of 13 cases versus 2 of 16 cases, respectively), whereas cases which were judged to be serious were less frequent (4 of 13 cases versus 14 of 16 cases, respectively).

#### Perceived causes

Mothers and witnesses mentioned a number of causes for the observed symptoms. In order of frequency these included fallen uvula – a condition involving a red and swollen uvula and resulting in inability to breastfeed and fever (28%, Amhara only), prolonged labor and common cold (17% each), pregnancy workload and poor hygiene as well as supernatural causes such as God, evil eye or evil spirits (10% each). Causes mentioned in less than 10% of cases included maternal conditions such as bleeding, abdominal cramping, HIV, poor diet or malnutrition, eating bad food, physical sprain and maternal cough, as well as exposure to environmental and metaphysical elements resulting in a local illness known as *mitch* and the use of a scented soap for bathing, evil spirits or pre-term birth resulting in an illness known as *tilla*. Causes also included newborn conditions such as being in a bad position (e.g., breech), being malnourished (e.g., due to twins), being born too soon, having the umbilical cord around the neck as well as improper cord tying. The causes mentioned by mothers and caregivers in 90% of cases might be considered as “physical” or “biological.”

Symptoms were sometimes thought to have multiple causes. For example, prolonged labor was associated with maternal twins or a baby that was in a bad position. In turn, prolonged labor was seen by some as a cause for inability to breast feed, continuous crying, difficult breathing or a cold body. A baby born too soon was associated with supernatural forces. Among some Amhara respondents, symptoms such as an inability to breastfeed, vomiting and fever were attributed to a swollen uvula which was, in turn, thought to be a result of natural processes, a heavy workload during pregnancy, bodily sprain or exposure to cold. Among Oromo respondents, these symptoms were thought to be caused by exposure to *mitch*, inadequate diet or poor hygiene. A cough, difficulty breathing and fever were often attributed to a common cold which was, in turn, thought to be a result of exposure to cold weather, a bad smell or lack of hygiene. As one father described,

“I have found out that he had difficulty breathing, fever, coughing and vomiting. I thought the problem was a common cold due to the cold weather and the smell from the cattle we share our house with.” (Father, Oromia).

#### Illness recognition

**Fig. 2:**
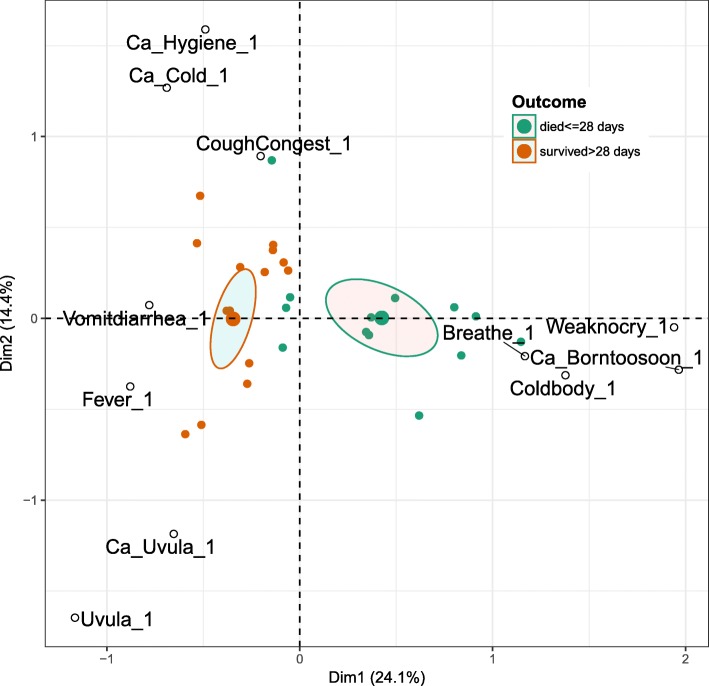
Multiple correspondence analysis of perceived symptoms, causes and outcomes

The MCA bi-plot map (Fig. 2) of the top 11 contributing variable levels for symptoms and causes reported by mothers and witnesses in each case shows that two MCA dimensions explained almost 40% (dimension 1 and 2 explains ~ 24 and 14%) of the variance in the data respectively. The cases are color-coded by two outcome groups: 1 = died within first 28 days, 2 = survived more than 28 days. One can see in the upper left quadrant of the map that symptoms of cough and congestion are correlated with causes common cold and poor hygiene, and that most of these newborns were among those that survived more than 28 days. Similarly, in the lower left quadrant one can see that symptoms of red swollen uvula and fever are associated the condition of fallen uvula, a folk category reported in Amhara only. In the lower right quadrant, symptoms of “weak or no cry,” “difficulty breathing,” “cold body,” and the cause “born too soon” were clustered together, and most of the newborns in this quadrant died within the first 28 days. The cases falling into the two outcome categories (marked by green and brown triangles) appear separated and associated with different kinds of symptoms and causes, and the 95% confidence ellipses drawn around the mean point of the two groups of individuals do not overlap, indicating that the group means were significantly different. There is heterogeneity in symptoms and causes among the cases in which the newborns survived.

#### Decision-makers and time to make a decision to seek care

Decision-makers included parents of the newborn as well as other close members of the family such as their parents, a sister, sister-in-law, daughter or neighbor. Although the decision to seek biomedical care was often made in consultation with others, the final decision-makers were often the parents (8 out of 29 cases) or the mother herself (8 cases). Fathers were less often the sole decision maker (4 cases). As one woman commented:

The baby spent the night crying a lot. It got even worse at night. I then decided that I would take the baby to the health center. Neighbors were complaining that I should not go to the health center before the baby was baptized. They said that an evil spirit would attack me. I ignored them and took the baby to the health center. (Amhara mother).

Of the 16 cases in which biomedical care was pursued, the time from illness recognition to the decision to seek care varied, from less than 12 h (six cases), 12–24 h (five cases), 25–72 h (two cases) togreater than 72 h (three cases, on days 5, 7 and 28).

### Delay 2

#### Care seeking pathways

Mothers and families took different pathways to access care for their newborns. Of the 16 newborns who survived the illness event, twelve families (75%) sought biomedical care (Table 1). Two newborns first received care at a health center. Seven newborns were first treated at home or by a local healer and secondly received care at a hospital, health center, health post or by an HEW who was called-in to the home. One of these newborns was subsequently treated at home and at a health center as third and fourth steps of care. Three newborns received two treatments at home or by a local healer who was called-in before being taken to a health center. One of them was also subsequently treated by a local healer who was called-in as a first step of care. Of the four surviving newborns who did not receive biomedical care (25%), two received treatment at home and two were treated by a local healer who was called-in. One of the former was also subsequently treated by a local healer.

**Table 1:** Biomedical and non-biomedicalcare-seeking steps taken by families of 16 surviving newborns

First Step	Second Step	Third Step	Fourth Step
Biomedical care Health Center (*n* = 2)			
Home	Hospital/Health Center/Post	Home	Health Center
(*n* = 5)	(*n* = 5)	(*n* = 1)	(*n* = 1)
Home	Home	Health Center	
(*n* = 1)	(*n* = 1)	(*n* = 1)	
Home	Home Call-in	Health Center	Home Call-in
(*n* = 2)	(*n* = 2)	(*n* = 1)	(*n* = 1)
Home Call-in	Hospital	Health Center	
(*n* = 1)	(*n* = 1)	(*n* = 1)	
Home Call-in	Home	Health Center	
(*n* = 1)	(*n* = 1)	(*n* = 1)	
Non-Biomedical care			
Home	Home Call-in		
(*n* = 2)	(*n* = 1)		
Home Call-in			
(*n* = 2)			

Of the 13 newborns who died, ten families (77%) did not seek biomedical care and were treated or cared for only at home (Table 2). Of the remaining three newborns, two received treatment at a health center and one at a hospital.

**Table 2:** Biomedical and non-biomedicalcare-seeking steps taken by families of 13 newborns who died

First Step	Second Step	Third Step	Fourth Step
Biomedical care			
Health Center			
(*n* = 2)			
Hospital			
(*n* = 1)			
Non-Biomedical care			
Home			
(*n* = 10)			

For analytical purposes, we chose to define care seeking at a health facility as a first or second step in response to newborn illness symptoms as ‘timely biomedical care seeking’. According to our definition, 12 of 29 families (41%) sought timely biomedical care in the neonatal period.

Timely biomedical care seeking was more frequent in the case of newborns who survived (8 of 16) compared to newborns who died (4 of 13). It was also associated with some characteristics of families. Timely biomedical care seeking was more frequent among younger mothers aged 19 to 29 years of age versus mothers older than 29 years of age (8 of 17 versus 3 of 10, respectively), mothers who delivered in a health facility as opposed to in a home or on the way to a health facility (10 of 18 versus 2 of 11, repectively), and, to a lesser extent, those who perceived the illness episode to be serious as compared to those who perceived it to be initially or ultimately not serious or hopeless (8 of 12 versus 10 of 17, respectively).

Symptoms and causes that appeared to have triggered biomedical care seeking were coughing, sneezing or stuffy nose associated with the common cold and poor maternal hygiene; and prolonged labor associated with maternal cramping and bleeding. Other symptoms included continuous crying, vomiting, malnutrition and improper cord tying. Symptoms that were as likely to trigger care seeking as not were difficulty breastfeeding and fever, both associated with a number of causes. Finally, symptoms and causes that were not associated with biomedical care seeking included swollen red uvula associated with the folk illness fallen uvula; and difficulty breathing, weak or no crying, and cold body associated with being born too soon or to supernatural causes. Few families relied on local traditional healers and birth attendants as a first or second step of care (6 of 29), in almost all cases for a fallen uvula.

### Facilitators, delayers and barriers to care seeking

Respondents reported a number of factors that facilitated, delayed or prevented their use of health facility care. Facilitators included physical and financial accessibility of HEWs or health posts, as well as health education and advice from HEWs, health workers or neighbors. Factors that either delayed or prevented care seeking included postpartum restrictions on women’s movement, perceived physical or spiritual vulnerability and weakness of post-natal women and newborns, hopelessness, the hope that the baby will get better, fear of travelling during the day time which may expose one to *mitch*, the evil eye or curious neighbors, or fears of poor treatment at the health facility. Other delaying factors were clinic hours, rain or night time hours, poor communications, distance, lack of transportation and financial constraints, e.g., one family that had to wait several weeks to receive a loan from their funeral association to cover medical costs.

## Discussion

### Summary of findings

In relation to Delay 1, the study findings suggest that the mothers and witnesses did recognize certain illness symptoms in their newborn. On the one hand, a number of symptoms were reported in clusters and were associated with particular causes such as being born too soon (premature), a common cold or lack of hygiene, or a fallen uvula. On the other hand, frequently mentioned symptoms such as difficulty breastfeeding and fever, important danger signs from a biomedical perspective, were associated with a variety of causes. Although most mothers and witnesses considered the symptoms they observed to be serious, some considered them not serious or only gradually came to believe they were serious, which led to a wait-and-see approach. As mentioned previously, more caretakers who considered their newborns’ illnesses symptoms to be hopeless or not serious were among those whose newborns died.

Symptoms or causes perceived as serious, however, did not necessarily lead to care seeking, as evident in the 10 families who did not seek biomedical care or sought late care. It is especially concerning that symptoms such as difficulty breastfeeding, fever, difficulty breathing, weak or no crying, and a cold newborn body did not trigger care seeking in all cases. Although the newborn’s parents were the main decision makers, others such as their parents, a sister, sister-in-law, daughter or neighbor were often involved. Their considerations about whether to seek care are consistent with the Delay Model [4] and include cultural norms such as perceived vulnerability and postpartum restrictions on the movement of mothers and newborns, advice from health workers, accessibility of services and perceived quality of care, as well as environmental conditions, economic and logistical issues.

In relation to Delay 2, in spite of the above considerations, 12 of 29 families sought timely bio-medical care, most often after an initial attempt at home-based care. Importantly, 38% of these families made the decision to seek care on the day that they recognized their newborn’s illness. And few families relied on local traditional healers and birth attendants as a first or second step of care (10% each), primarily for the traditional illness, fallen uvula. Timely biomedical care seeking was more common among younger mothers, mothers who had delivered in a health facility, babies who survived the neonatal period, and to some extent among families who perceived the illness episode to be serious.

Researchers examining illness recognition and care seeking typically conduct descriptive studies using mixed methods and situated in a variety of settings, often in South Asia and sub-Saharan Africa. Some of these studies found that newborn caretaker recognition of newborn illness symptoms to be poor [9, 10] whereas other studies found that care takers did recognize such symptoms [11, 12]. Findings from our study conform with the latter including symptom clusters associated with the common cold, the folk illness fallen uvula, and preterm babies and those with difficulty breathing, which are associated with different causes and outcomes. Previous studies have shown that perceived causes of newborn illness range from the supernatural to naturalistic which influence whether traditional or modern treatment is utilized [12, 13, 14]. In this study, illness responses exhibited some association with specific types of mostly naturalistic causes. Conceptions and responses related to the fallen uvula illness in Amhara region resemble the local illnesses recognized in other countries which are seen to be best treated with traditional medicine [10, 15]. Illness symptoms that are considered serious have been found to be associated with care seeking in some studies whereas this association was weaker in a study conducted in Ghana and also in our study [10, 16].

Use of biomedical care ranging from 14 to 39% of illness episodes have been reported in various studies as compared to 40% in this study [11, 17, 18]. One of these studies conducted in Nepal reported that half of those who sought medical care did so after the first 48 h from the onset of illness as compared to 38% who sought such care in the first day after symptoms were recognized in our study [14]. The data we have presented on the characteristics of newborn care takers who seek such care are often not available in similar studies and contrast with the findings of one study on characteristics associated with care seeking for children under five [19]. We have also found that newborn survival is associated with facility delivery, the assessment that illness symptoms are serious and with the use of biomedical care.

Previous research has also explicitly identified factors which delay or prevent care seeking for newborn illnesses. Barriers such as aspects of local understanding of illness including symptom recognition, causation and severity, associated use of traditional treatments, wait-and-see attitudes, hopelessnes, negative experiences at health facilities, and lack of physical and financial access have been discussed in various studies [10, 14, 17, 20]. While finding that symptom recognition is not as much of a constraint on care seeking, this study has also identified all these barriers in addition to the role of postpartum restrictions on the movement and perceived vulnerability of women and newborns, reluctance to travel during the day time, limited clinic hours, and environmental factors. Furthermore, enablers such as the physical and financial accessibility of health posts and health counseling and education from health workers and acquaintances have been identified.

### Strengths and challenges

The illness narrative method generated data on the actual experience and diverse perspectives of witnesses to the event. The illness event timeline and neutral probes used in the narrative interview stimulate recall and increase validity of the data. The replicability of the narrative interviews is unknown.

## Conclusions

The findings of this study show that mothers and other witnesses generally recognize certain newborn illness symptoms and their seriousness. Several of them did initially or ultimately consider symptoms to be not serious or hopeless. Recognition of the seriousness of symptoms does not always lead to timely biomedical care seeking, although in our setting care seeking appears to occur more frequently among younger mothers, as well as those who gave birth in a health facility. The findings thus indicate an urgent need to focus health education and behavior change efforts on the seriousness as well as treatability of illness symptoms and identified local cultural factors that impede care seeking such as traditional postpartum restrictions on women’s movement and associated beliefs about the vulnerability of mothers and newborns to harmful metaphysical elements if taken outside of the home, hopelessness and, in the case of Amhara region, the folk illness, fallen uvula. Families must come to understand timely biomedical care will improve the chances of their newborns’ survival. Continued efforts to reduce known environmental, logistic and economic barriers to care seeking are also needed.

## Abbreviations

FMoH: Federal Ministry of Health
HDA: Health Development Army
HEW: Health Extension Worker
MaNHEP: Maternal and Newborn Health in Ethiopia Partnership
RHB: Regional Health Bureau
URC: University Research Company, LLC
USAID: United States Agency for International Development
